# Trends in patient characteristics and clinical outcome over 8 years of transcatheter aortic valve implantation

**DOI:** 10.1007/s12471-018-1129-x

**Published:** 2018-06-25

**Authors:** F. van Kesteren, M. S. van Mourik, E. M. A. Wiegerinck, J. Vendrik, J. J. Piek, J. G. Tijssen, K. T. Koch, J. P. S. Henriques, J. J. Wykrzykowska, R. J. de Winter, A. H. G. Driessen, A. Kaya, R. N. Planken, M. M. Vis, J. Baan

**Affiliations:** 1 Heart Centre, Academic Medical Centre, University of Amsterdam, Amsterdam, The Netherlands; 2 Department of Radiology and Nuclear Medicine, Academic Medical Centre, University of Amsterdam, Amsterdam, The Netherlands

**Keywords:** Transcatheter aortic valve implantation, Transcatheter aortic valve replacement, Outcome, Mortality, Survival

## Abstract

**Aim:**

In the evolving field of transcatheter aortic valve implantations (TAVI) we aimed to gain insight into trends in patient and procedural characteristics as well as clinical outcome over an 8‑year period in a real-world TAVI population.

**Methods:**

We performed a single-centre retrospective analysis of 1,011 consecutive patients in a prospectively acquired database. We divided the cohort into tertiles of 337 patients; first interval: January 2009–March 2013, second interval: March 2013–March 2015, third interval: March 2015–October 2016.

**Results:**

Over time, a clear shift in patient selection was noticeable towards lower surgical risks including Society of Thoracic Surgeons predicted risk of mortality score and comorbidity. The frequency of transfemoral TAVI increased (from 66.5 to 77.4%, *p* = 0.0015). Device success improved (from 62.0 to 91.5%, *p* < 0.0001) as did the frequency of symptomatic relief (≥1 New York Heart Association class difference) (from 73.8 to 87.1%, *p* = 0.00025). Complication rates decreased, including in-hospital stroke (from 5.0 to 2.1%, *p* = 0.033) and pacemaker implantations (from 10.1 to 5.9%, *p* = 0.033). Thirty-day mortality decreased (from 11.0 to 2.4%, *p* < 0.0001); after adjustment for patient characteristics, a mortality-risk reduction of 72% was observed (adjusted hazard ratio [HR]: 0.28, 95% confidence interval [CI]: 0.13–0.62). One-year mortality rates decreased (from 23.4 to 11.4%), but this was no longer significant after a landmark point was set at 30 days (mortality from 31 days until 1 year) (adjusted HR: 0.69, 95% CI: 0.41–1.16, *p* = 0.16).

**Conclusion:**

A clear shift towards a lower-risk TAVI population and improved clinical outcome was observed over an 8‑year period. Survival after TAVI improved impressively, mainly as a consequence of decreased 30-day mortality.

**Electronic supplementary material:**

The online version of this article (10.1007/s12471-018-1129-x) contains supplementary material, which is available to authorized users.

## What’s new

- In recent years, there has been a clear shift towards a lower-risk transcatheter aortic valve implantation (TAVI) population.
- Over the last few years, TAVI has become a significantly safer treatment.
- Survival after TAVI has improved, mainly as a consequence of decreased 30-day mortality.

## Introduction

Since the first transcatheter aortic valve implantation (TAVI) was performed in 2002, the procedure has become well-established for the treatment of patients with severe symptomatic aortic valve stenosis [1, 2]. The safety and efficacy of TAVI were first established for patients who were inoperable or at high risk for surgical aortic valve replacement (SAVR) [3–5]. Influenced by these results, the number of procedures has increased [6]. Subsequent studies revealed similar outcomes for TAVI and SAVR in intermediate-risk patients [7–11]. But although these results are promising, trials only serve as a benchmark for clinical practice, and it is not known how they affect treatment in a real-world setting. It is also not clear if there is an actual shift in the TAVI population towards patients with lower surgical risks. In addition, over the years the prostheses and the procedure have been refined and increasing experience in centres and among operators may have influenced the outcome of TAVI. Therefore, we aimed to gain insight into the trends in patient and procedural characteristics as well as clinical outcome after TAVI in a consecutive single-centre, real-world population over an 8‑year period beginning in 2009.

## Methods

### Patient population

We used the data of a prospectively acquired population that comprised 1,011 consecutive patients who underwent TAVI between 8 January 2009 and 26 October 2016 at the Academic Medical Centre Amsterdam, The Netherlands. Cardiologists and cardiac surgeons in the heart team and multidisciplinary TAVI team discussed the information of all patients. As of June 2011 a radiologist was added to the team and in February 2016 a geriatrician followed. A cardiac anaesthesiologist reviewed the patients separately. The team made the decision to select patients for TAVI rather than SAVR based on a combination of age, condition and medical history. If a patient was denied surgery, the team evaluated her/his eligibility for TAVI. If there was any uncertainty about denying surgery, patients were reviewed at the outpatient clinic once more. The default TAVI approach was transfemoral. If small vessel diameters or abnormalities precluded this access, patients underwent transapical or transaortic TAVI. Device type and sizing was at the discretion of the operators, based on measurements performed at pre-operative screening and available devices at that time.

### Outcome

In compliance with national legislation, the Institutional Review Board approved this study and provided a waiver for the retrospective analysis of data. To enable a statistically balanced comparison, the cohort was divided into equal tertiles. All tertiles contained 337 patients: first procedural time interval from 8 January 2009 to 3 March 2013; second interval from 4 March 2013 to 3 March 2015; and third interval from 3 March 2015 to 26 October 2016. Baseline patient and procedural characteristics were documented. Society of Thoracic Surgery predicted risk of mortality (STS-PROM) scores were calculated online (V2.81 in use since 2014). Because of the small number of patients that underwent transaortic and transapical TAVI, we combined them as a transthoracic cohort. Clinical outcome was scored according to the Valve Academic Research Consortium (VARC)-2 consensus document [12]. Mortality data were obtained via the centralised Dutch national municipal population register in January 2017, ensuring complete survival follow-up. Information on causes of death was obtained from the hospitals’ records or primary care physicians.

### Statistical analysis

Categorical variables were compared using a chi-square test for trend. Continuous data were compared using one-way ANOVA or the non-parametric Jonckheere Terpstra trend test as appropriate. Survival distributions were compared according to Kaplan Meier, using log-rank for linear trend. We tested the proportional hazard assumption with log-minus-log plots and Schoenfeld residuals. Because the assumption was violated we performed landmark analyses. With landmark analysis, follow-up time is divided into periods of interest with a landmark point. Patients whose survival is shorter than this point are excluded from subsequent analyses [13]. Based on the survival curves and clinical perspective we set the landmark at 30 days. Univariate and multivariate Cox proportional hazard models were used to calculate (un)adjusted hazard ratios (HR) at the landmark point of 30 days and at 1 year (HR from 31 days to 1 year). To determine covariates included in multivariate analysis, we performed univariate analyses for all patient-related baseline characteristics and procedural approach. For the multivariate analysis, we adjusted for covariates with a *p*-value <0.20 at univariate analyses. We used multiple imputations to address missing data accounted for in Cox regression. Data on left ventricular ejection fraction, right ventricular function, systolic pulmonary artery pressure and mitral regurgitation grade were missing in 0.3%, 0.5%, 2.6% and 0.8% of the patients, respectively. We assumed data were missing at random. By using the fully conditional specification approach based on a model incorporating clinical characteristics, five datasets were created. Pooled results of these datasets were used. A *p*-value <0.050 was considered significant. Analyses were performed on SPSS version 23.0 (IBM corporation, Chicago, IL, USA) and the *survival* package in R‑statistical software version 3.3.1 [14].

## Results

### Patient characteristics

**Fig. 1:**
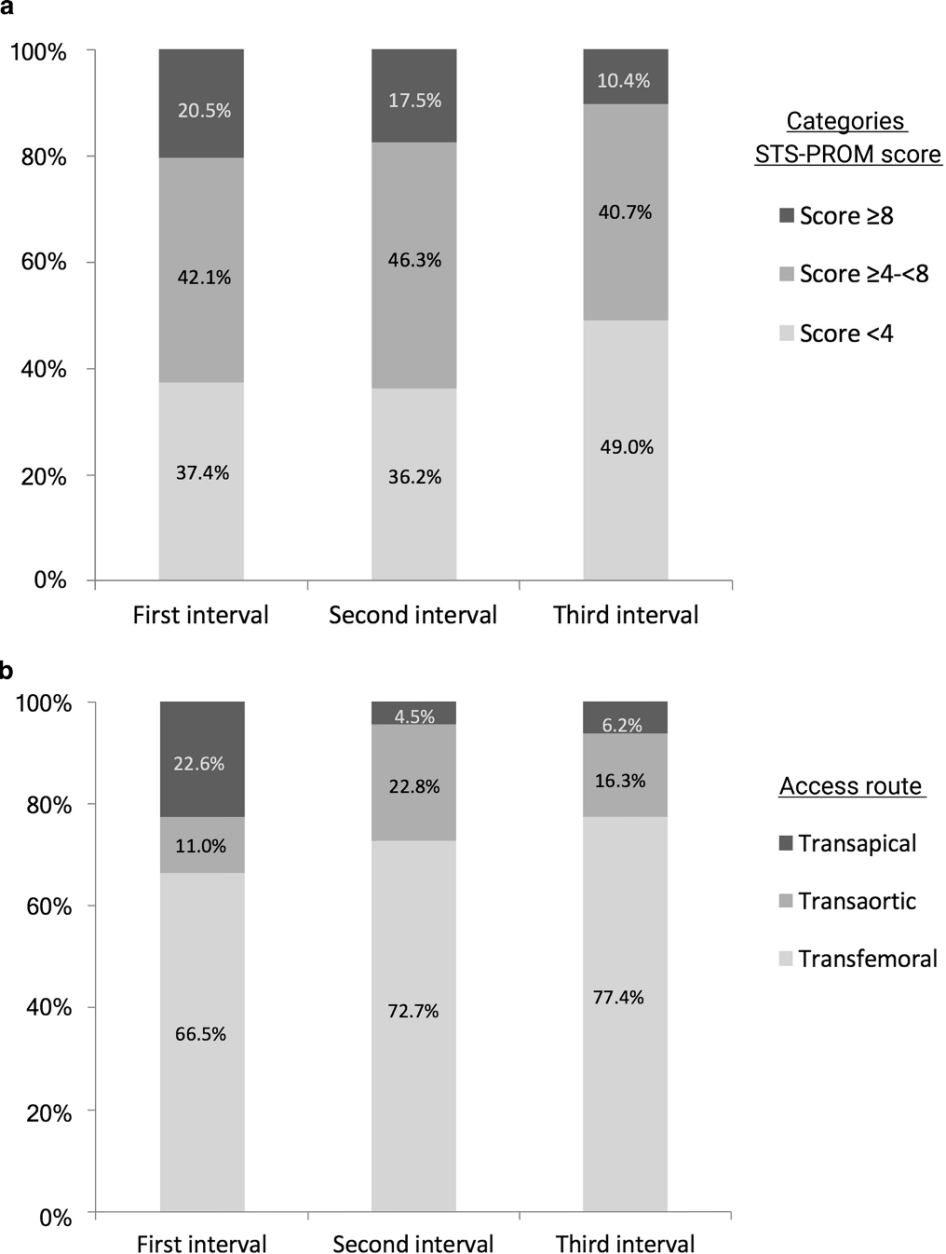
Distribution of Society of Thoracic Surgery predicted risk of mortality (*STS-PROM*) scores (**a**) and access routes (**b**) per procedural time interval. STS-PROM scores divided into categories: low risk <4, intermediate ≥4–<8 and high ≥8. Numbers are the percentages of patients within the procedural time interval

Patient characteristics per procedural time interval are described in Tab. 1. The relative number of patients who underwent TAVI with a low STS-PROM score (<4) increased from 37.4% in the first to 49.0% in the third interval (Fig. 1a). Patients in the third interval had comorbidities significantly less often (Tab. 1). The number of procedures performed in an urgent (not-elective) setting decreased from 24.0% in the first to 11.9% in the third interval (*p* < 0.001).

**Table 1:** Demographic and clinical characteristics per procedural time interval

Characteristics	Time interval			*p*-value
	First interval (*n* = 337)	Second interval (*n* = 337)	Third interval (*n* = 337)	
Age (years)	82 (77–85)	82 (77–85)	81 (77–85)	0.44
Male sex	133 (39.5%)	150 (44.5%)	157 (46.6%)	0.062
Body mass index (kg/m^2^)	26.7 (24.2–30.6)	26.7 (24.1–30.2)	26.9 (24.0–30.0)	0.57
NYHA class III or IV	249 (73.9%)	239 (70.9%)	234 (69.4%)	0.20
Urgent setting	81 (24.0%)	85 (25.2%)	40 (11.9%)	<0.0001
*Cardiovascular medical history*				
– Hypertension	264 (78.3%)	293 (86.9%)	275(81.6%)	0.27
– Atrial fibrillation	120 (35.6%)	144 (42.7%)	139 (41.2%)	0.14
– Valvular surgery	12 (3.6%)	13 (3.9%)	13 (3.9%)	0.84
– Coronary bypass surgery	60 (17.8%)	43 (12.8%)	38 (11.3%)	0.014
– PCI	107 (31.8%)	95 (28.2%)	80 (23.7%)	0.020
– Pacemaker	28 (8.3%)	41 (12.2%)	32 (9.5%)	0.61
– Stroke	32 (9.5%)	45 (13.4%)	34 (10.1%)	0.81
– Peripheral artery disease	106 (31.5%)	96 (28.2%)	90 (26.7%)	0.17
*Non-cardiovascular medical history*				
– COPD	134 (39.8%)	117 (34.7%)	83 (24.6%)	<0.0001
– Diabetes mellitus	102 (30.3%)	104 (30.9%)	110 (32.6%)	0.51
– Renal clearance <60 ml/min	145 (43.0%)	145 (43.0%)	133 (39.5%)	0.35
– Liver cirrhosis	6 (1.8%)	9 (2.7%)	3 (0.9%)	0.38
STS-PROM (score)	4.838 (3.315–7.176)	4.833 (3.273–6.868)	4.022 (2.830–5.947)	0.00013
*STS-PROM categories*				<0.0001
– STS-PROM score <4	126 (37.4%)	122 (36.2%)	165 (49.0%)	–
– STS-PROM score ≥4 <8	142 (42.1%)	156 (46.3%)	137 (40.7%)	–
– STS-PROM score ≥8	69 (20.5%)	59 (17.5%)	35 (10.4%)	–
Euroscore I (score)	16.19 (10.34–25.72)	14.70 (10.11–22.22)	12.87 (9.07–20.12)	<0.0001
Euroscore II (score)	4.69 (2.82–8.28)	4.31 (2.69–7.44)	3.73 (2.33–6.50)	<0.0001
Porcelain aorta	23 (6.8%)	21 (6.2%)	11 (3.3%)	0.042
Hostile chest	26 (7.7%)	25 (7.4%)	24 (7.1%)	0.77
*Pre-procedural echocardiography*				
– LVEF <30%	30/335 (9.0%)	24/337 (7.1%)	26/336 (7.7%)	0.56
– Moderate/severe RVF	18/335 (5.4%)	20/336 (6.0%)	26/335 (7.8%)	0.21
– SPAP >55 mm Hg	45/331 (13.6%)	28/327 (8.6%)	24/327 (7.3%)	0.0070
– Moderate/severe MR	46/336 (13.7%)	39/334 (12.3%)	26/333 (7.8%)	0.016

If data were missing denominators are notated

*NYHA* New York Heart Association, *PCI* percutaneous coronary intervention, *COPD* chronic obstructive pulmonary disease, *STS-PROM* Society of Thoracic Surgery predicted risk of mortality, *LVEF* left ventricle ejection fraction, *RVF* right ventricle failure, *SPAP* systolic pulmonary artery pressure, *MR* mitral regurgitation

### Procedural characteristics

**Fig. 2:**
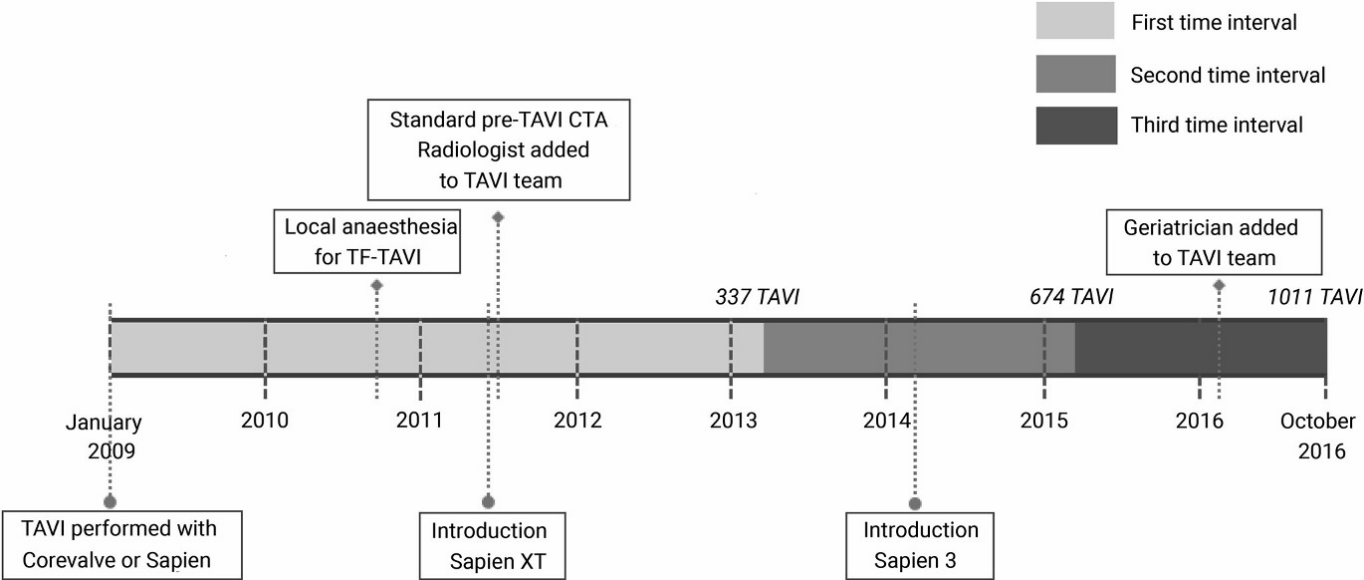
Timeline of the TAVI programme at the Academic Medical Centre Amsterdam, The Netherlands. (*CTA* computed tomography angiography, *TAVI* transcatheter aortic valve implantation, *TF-TAVI* transfemoral TAVI)

The number of TAVIs per year increased from 81 in the first interval to 169 and 204 in the second and third interval, respectively. Over time, 730 transfemoral procedures were performed (72.2%) with an increasing frequency over time (Fig. 1b; Tab. 2). Of the 281 patients with transthoracic TAVI, 169 underwent transaortic (60.1%) and 112 transapical procedures (39.9%). The number of transaortic procedures increased compared to transapical procedures (Fig. 1b). As demonstrated in Fig. 2, changes in the TAVI program were carried out. The number of procedures performed with balloon-expandable devices increased and as new prostheses were introduced there was a clear shift in the devices used (Fig. 2; Tab. 2). The operator team increased from seven to nine operators, with five operators performing TAVI in all intervals; two operators left the team (first and second interval) and four new operators joined (three in the second and one in the third interval). The number of procedures per operator increased, but this was not significant (Tab. 2).

**Table 2:** Procedural characteristics per procedural time interval

Characteristics	Time interval			*p*-value
	First interval (*n* = 337)	Second interval (*n* = 337)	Third interval (*n* = 337)	
Access transfemoral	224 (66.5%)	245 (72.7%)	261 (77.4%)	0.0015
– of whom general anaesthesia	55/224 (24.6%)	8/245 (3.3%)	3/261 (1.1%)	<0.0001
Access transthoracic	113 (33.5%)	92 (27.3%)	76 (22.6%)	
– of whom transaortic	37/113 (32.7%)	77/92 (83.4%)	55/76 (72.4%)	<0.0001
Prosthesis balloon expandable	197 (58.5%)	334 (99.1%)	318 (94.4%)	<0.0001
– Medtronic Corevalve	140 (41.5%)	2 (0.6%)	–	
– Edwards Sapien	83 (24.6%)	11 (3.3%)	–	
– Edwards Sapien XT	113 (33.5%)	128 (38.0%)	4 (1.2%)	
– Edwards Sapien 3	–	195 (57.9%)	310 (92.0%)	
– Other	1 (0.3%)	1 (0.3%)	23 (6.8%)	
*Operator experience*				
– Procedures/operator/year	33 (29–45)	35 (21–79)	44 (23–70)	0.60
– Number of operators	7	9	9	
– Cumulative procedures/operator				0.44
a. <50 procedures	2/7 (28.6%)	3/9 (33.3%)	1/9 (33.3%)	
b ≥50 <100 procedures	1/7 (14.3%)	1/9 (11.1%)	3/9 (44.4%)	
c. ≥100 <250 procedures	4/7 (57.1%)	2/9 (22.2%)	2/9 (22.2%)	
d. ≥250 procedures	–	3/9 (33.3%)	3/9 (33.3%)	

Transthoracic access consisted of patients undergoing transaortic and transapical transcatheter aortic valve implantation

### Outcome

Procedural outcome is described in Tab. 3. Although major vascular and bleeding complications decreased numerically, the changes were not significant (6.5 to 3.6% [*p* = 0.10] and 9.2 to 5.6% [*p* = 0.096]).

**Table 3:** Procedural outcome after transcatheter aortic valve implantation per procedural time interval

Characteristics	Time interval			*p*-value
	First interval (*n* = 337)	Second interval (*n* = 337)	Third interval (*n* = 337)	
NYHA decreased ≥1 point^a^	186/252 (73.8%)	206/246 (83.7%)	175/201 (87.1%)	0.00025
Device success^b^	204/329 (62.0%)	288/332 (86.7%)	303/331 (91.5%)	<0.0001
*In-hospital complications*				
– Stroke	17 (5.0%)	10 (3.0%)	7 (2.1%)	0.033
– Major vascular complication	22 (6.5%)	25 (7.4%)	12 (3.6%)	0.10
– Major bleeding	31 (9.2%)	35 (10.4%)	19 (5.6%)	0.096
– New pacemaker implantation	35 (10.4%)	26 (7.7%)	20 (5.9%)	0.033

If data were missing denominators are notated

*NYHA* New York Heart Association class

^a^Measured at 30–60 days after the procedure

^b^Defined as the composite end-point according to VARC-2 criteria: absence of 30-day mortality, correct positioning of a single prosthesis and prosthesis performance

**Fig. 3:**
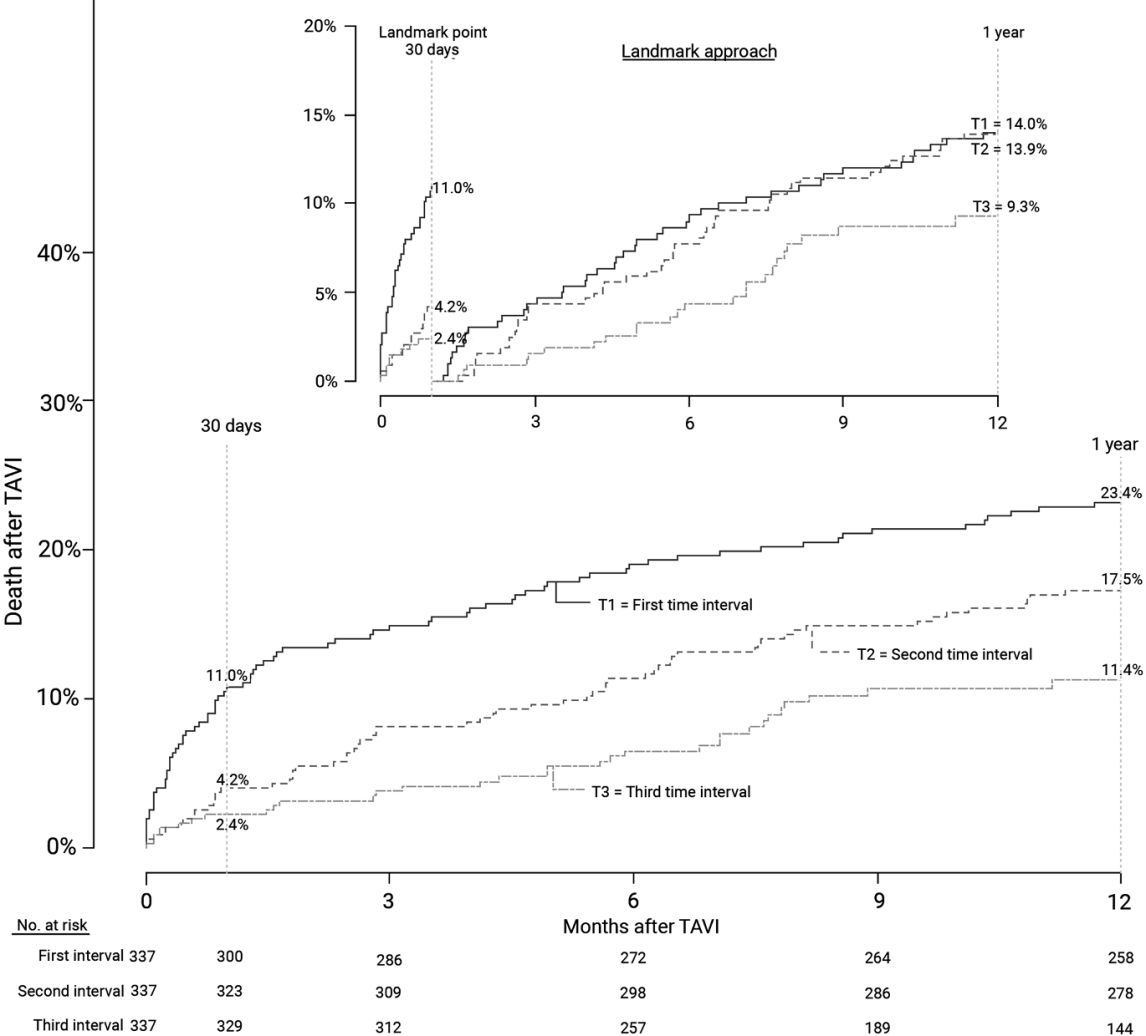
Time to event curve—death from any cause since TAVI. Mortality rates per procedural time interval. Numbers are the cumulative incidence estimates at the landmark point at 30 days and 1 year. The *inset* shows the analysis with a landmark approach. (*TAVI* transcatheter aortic valve implantation)

**Fig. 4:**
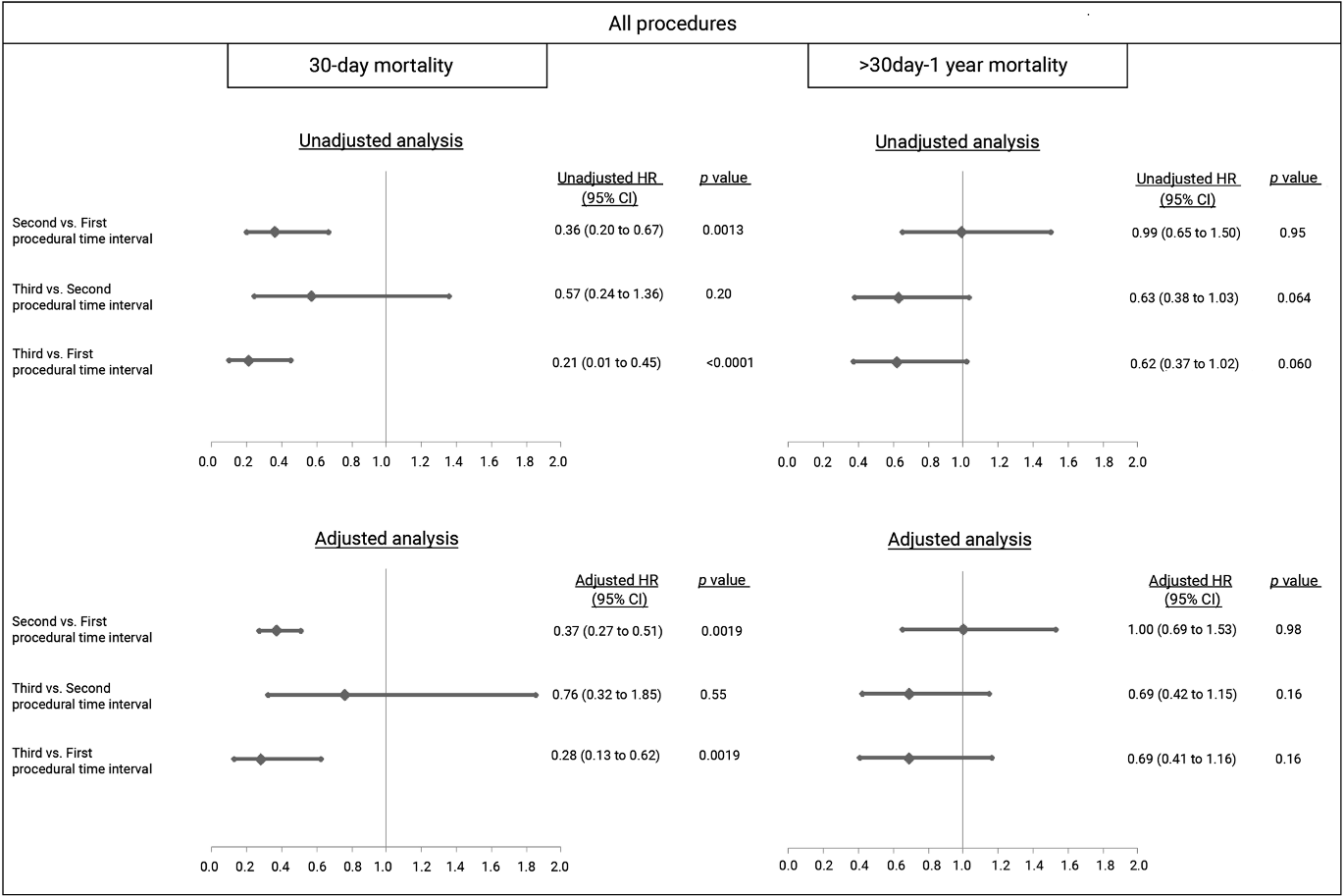
Adjusted hazard ratios (*HR*) for all-cause mortality after TAVI, landmark approach. HR at the landmark point at 30 days and after the landmark at 1 year, comparison per procedural time interval. Adjustment at 30 days for gender, body mass index (BMI), New York Heart Association (NYHA) class, valvular surgery, percutaneous coronary intervention (PCI), peripheral artery disease, chronic obstructive pulmonary disease (COPD), diabetes, Society of Thoracic Surgery predicted risk of mortality (STS-PROM) score, left ventricular ejection fraction (LVEF), mitral regurgitation (MR), urgent and transthoracic procedure. Adjustment at 1 year for gender, BMI, NYHA, valvular surgery, coronary artery bypass graft, COPD, diabetes, estimated glomerular filtration rate, STS-PROM score, hostile chest, LVEF, right ventricle failure, systolic pulmonary artery pressure, MR, urgent and transthoracic procedure

Thirty-day mortality decreased from 11.0% in the first to 4.2 and 2.4% in the second and third interval, respectively (*p* = <0.0001; Fig. 3). After adjustment, TAVI in the third interval was associated with a decrease in 30-day mortality of 72% compared to the first interval (adjusted HR: 0.28 [95% CI 0.13–0.62] *p* = 0.0019; Fig. 4). At 1 year, mortality still differed with rates of 23.4% in the first and 17.5 and 11.4% in the second and third interval (*p* = <0.0001). However, with landmark analysis from 30 days until 1 year, the difference became non-significant (*p* = 0.071; Fig. 3).

For transfemoral TAVI, 30-day mortality significantly decreased (first, second, third interval: 8.9%, 2.5%, 1.2%; *p* = <0.0001). At 1 year, mortality rates still differed: 19.2, 15.5 and 12.1% (*p* = 0.016), an effect that did not continue to exist after landmark analysis (*p* = 0.89; Supplementary Fig. S1). For transthoracic TAVI no significant difference was found in 30-day mortality between the intervals (first, second, third: 15.0%, 8.7%, 6.6%; *p* = 0.052). However, at 1 year the rates per interval did differ (first, second, third: 31.9%, 22.8%, 9.7%; *p* = 0.00066), and remained significant after performing landmark analysis (*p* = 0.0044; Supplementary Fig. S1).

Specific causes of death are described in Supplementary Tab. S1. Of all 170 patients who died within 1 year, 138 deaths were of cardiac origin (81.2%). At 1 year, heart failure was described in most patients as the primary cause of death (48 patients; 28.2% of all deaths), followed by stroke (21 patients; 12.4%). Of the non-cardiovascular causes of death, malignancy was described most often (15 patients; 8.8%).

## Discussion

Over an 8‑year period we demonstrate a definite shift in the TAVI population towards patients with lower surgical risks and less comorbidity, as well as changes in procedural characteristics and significantly better clinical outcome. By the use of landmark analyses we discriminated between trends in mortality until 30 days and thereafter. The most striking decline in mortality was seen within 30 days, as even after adjustment for the changing population the mortality risk decreased by 72% in the most recent time interval compared to earlier years (2015–2016 vs. 2009–2013).

Studies reporting a good outcome in patients with decreasing surgical risks contributed to the shift in TAVI population [7–11]. The reported lower risk scores and comorbidities of our patients over the years are a reflection of this change in patient selection. It could be suggested that the shift towards lower-risk patients is the explanation for the decline in complication and mortality rates. But although this could have had an influence, with our adjusted analysis we demonstrated that this was not the only explanation. Devices underwent multiple refinements that in general favourably influenced outcome [15–17]. Most likely, the improved outcome is strongly influenced by increased operator and institutional experience combined with procedure- and device-related changes. The impact of this experience on outcome is difficult to quantify. We show that our care for TAVI patients became even more professional by the addition of a radiologist and a geriatrician to our TAVI team. In addition, the number of procedures performed per year increased. Most studies analysing learning curves describe proficiency to be reached after 25–50 TAVIs [18–20]. In our centre, experienced operators trained new operators and all performed more than 25 TAVIs, with the most experienced operator performing more than 500 procedures.

The relative increase in transfemoral procedures described in our cohort was in compliance with trends described elsewhere [21, 22]. Another mentionable aspect is that, unless prohibited for clinical reasons, since 2010 we have performed transfemoral TAVI under local anaesthesia. This leads to the rather unique situation in which only 1.1% of all transfemoral procedures are performed under general anaesthesia, while in the recently published Transcatheter Valve Therapy (TVT) registry more than three-quarters of the patients underwent general anaesthesia [21].

Noteworthy for the interpretation of our results are the low STS-PROM scores we report in all intervals. According to these scores more than half of our population has an ‘intermediate’ or even ‘low’ surgical risk. This may be explained in part by the fact that we (re-)calculated all risks by the latest version of the STS calculator. Reporting scores as calculated within their own interval might have given different results, but would not have allowed a legitimate comparison. Furthermore, it is important to be aware that the scores do not cover all aspects to determine procedural risk. In this context, it must be mentioned that in all intervals all patients were first denied SAVR. Nevertheless, the lower risk profile of our patients might have influenced our favourable outcome as compared to studies including only high-risk patients.

The mortality rates we describe resemble data of recently published registries; in the latest interval 30-day mortality was 2.4%, slightly lower than the results of the TVT registry (4.6%) and comparable with the rates of the low-risk OBSERVANT (2.5%) and SOURCE registry (2.2%) [21, 23, 24]. The 1‑year mortality we report (11.4%) was low compared to that of the TVT registry (21.6%) and similar to the OBSERVANT (11.4%) and the Asian TAVI registry (10.8%) [21, 23, 25]. Also in compliance with others we report initially higher mortality rates at 30 days for transthoracic compared to transfemoral TAVI [26, 27]. Nevertheless, outcome for transthoracic TAVI at 1 year significantly improved over the intervals.

As we demonstrated, 30-day mortality rates significantly declined, but mortality until 1 year did not show the same impressive trend. In the pursuit of further improving outcome, the focus should be on this later time window, e. g. by improved patient selection. We believe TAVI will continue to develop and expand. The introduction of new prostheses will further diminish procedural complications including vascular complications, regurgitation and hopefully pacemaker implantations and strokes. For the last complication, the focus of trials should be on optimised anticoagulation therapy. Moreover, long-term results are eagerly awaited to gain insight into prosthesis durability. The indication for TAVI might shift further to lower-risk populations. Lastly, it can be expected that the future will bring risk scores, evaluating procedural mortality and morbidity and perhaps tailored device selection, all optimising outcome and expectations after TAVI.

The present analysis was retrospectively conducted on a single-centre, non-randomised but prospectively acquired cohort and has therefore inherent limitations to such a design. Also the use of landmark analyses has limitations including omission of time-to-event distribution prior to the landmark point [28]. Although it is the real-world situation, the number of transthoracic TAVIs was relatively low, while this was already a combination of two separate approaches. In addition, not all patients in the third time interval had a complete 1‑year follow-up. However, median follow-up in this time interval was still 342 days. By the use of the Dutch population register, we were ensured that we had complete survival data, minimising censoring only to patients with ‘drop-out’ as a consequence of study time.

## Conclusion

Over an 8‑year period a clear shift towards a lower-risk population and significantly improved clinical outcomes was observed in a real-world TAVI cohort. Survival after TAVI improved impressively, mainly as a consequence of decreased 30-day mortality. Over time, TAVI has become a significantly safer treatment for patients with symptomatic aortic valve stenosis.

## Caption Electronic Supplementary Material

Supplemental Fig. S1 Time to event curve—death from any cause since TAVI per TAVI approach. Split time to event curves for transfemoral (**a**) and transthoracic (**b**) TAVI; comparison per procedural time interval. Numbers are cumulative incidence estimates at the landmark point at 30 days and 1 year. The *inset* shows the analysis with landmark approach. Transthoracic procedures comprise all transaortic and transapical TAVI

Supplemental Tab. S1 Causes of death per time interval of the procedure divided by the timing of death during follow-up
