# Supplementary figures and images for: Trends in patient characteristics and clinical outcome over 8 years of transcatheter aortic valve implantation
Source: Neth Heart J. 2018 Jun 25;26(9):445–53. doi: 10.1007/s12471-018-1129-x (PMC6115311; doi:10.1007/s12471-018-1129-x)

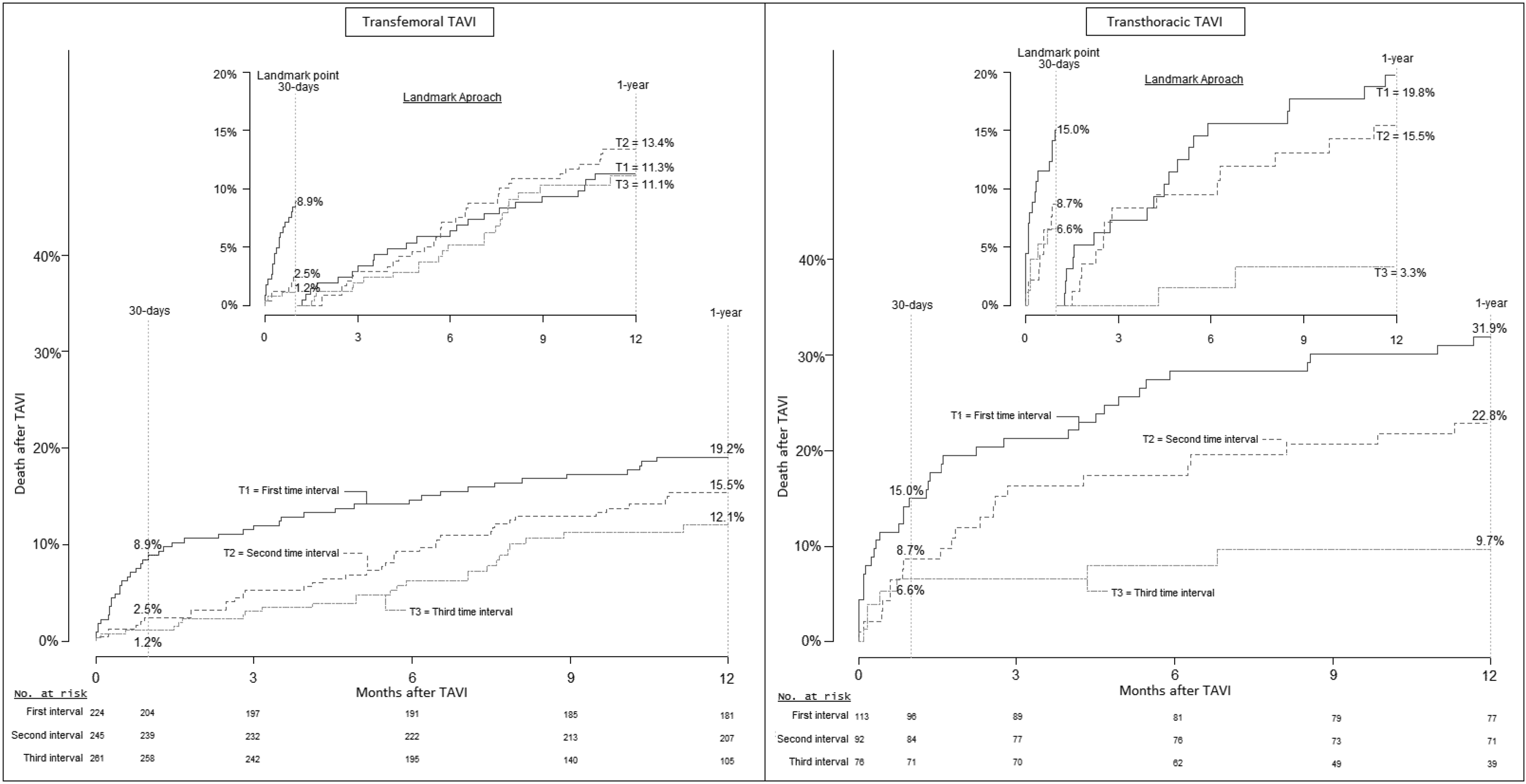

Supplement: Supplementary file 1 — Supplemental Fig. S1 Time to event curve—death from any cause since TAVI per TAVI approach. Split time to event curves for transfemoral (a) and transthoracic (b) TAVI; comparison per procedural time interval. Numbers are cumulative incidence estimates at the landmark point at 30 days and 1 year. The inset shows the analysis with landmark approach. Transthoracic procedures comprise all transaortic and transapical TAVI [file 12471_2018_1129_MOESM1_ESM.jpg]
