# Supplementary material for: Trends in patient characteristics and clinical outcome over 8 years of transcatheter aortic valve implantation
Source: Neth Heart J. 2018 Jun 25;26(9):445–53. doi: 10.1007/s12471-018-1129-x (PMC6115311; doi:10.1007/s12471-018-1129-x)
Supplement: Supplementary file 2 — Supplemental Tab. S1 Causes of death per time interval of the procedure divided by the timing of death during follow-up [file 12471_2018_1129_MOESM2_ESM.doc]

Supplemental table S1 Causes of death per time interval of the procedure divided by the timing of death during follow-up

|  | **Time interval** | | |
| --- | --- | --- | --- |
|  | **First interval** | **Second interval** | **Third interval** |
| **Cardiovascular death** |  |  |  |
| *≤72 hours* | n=337 | n=337 | n=337 |
| End-stage heart failure | 3 | - | - |
| Myocardial infarction | 2 | - | - |
| Arrhythmia | 3 | - | - |
| Tamponade | - | 2 | 1 |
| Hypovolemic shock (procedure related) | 4 | 1 | 1 |
| Stroke | 1 | - | - |
| Anaphylaxis | - | - | 1 |
| *>72 hours -≤30 days* | n=324 | n=334 | n=334 |
| End-stage heart failure | 8 | 4 | 2 |
| Myocardial infarction | 1 | - | 1 |
| Hypovolemic shock (procedure related) | 3 | 4 | - |
| Stroke | 6 | 1 | 1 |
| Intestinal ischemia (procedure related) | 1 | 1 | 1 |
| Infection; non-cardiac (procedure related) | 3 | 1 | - |
| Unwitnessed death/Unknown cause | 2 | - | - |
| *>30 days -1 year* | n=300 | n=323 | n=329* |
| End-stage heart failure | 12 | 15 | 4 |
| Myocardial infarction | 1 | - | 1 |
| Arrhythmia | - | 1 | 1 |
| Hypovolemic shock | 2 | 1 | - |
| Stroke | 5 | 6 | 1 |
| Endocarditis | 3 | 1 | 3 |
| Infection; non-cardiac (procedure related) | 2 | - | - |
| Critical limb ischemia | 1 | - | 1 |
| Clinical deterioration† | 2 | 1 | 1 |
| Unwitnessed death/Unknown cause | 5 | 5 | 4 |
| **Non-cardiovascular death** |  |  |  |
| *>30 days -1 year* | n=300 | n=323 | n=329* |
| Malignancy | 4 | 7 | 4 |
| Trauma | - | - | 3 |
| Infection (non-cardiac) | 3 | 6 | - |
| End-stage renal failure | 1 | 1 | - |
| Chronic pulmonary disease | 1 | 1 | - |
| Morbus Parkinson | - | - | 1 |

*with a median follow-up of 30 + 312 days. †Clinical deterioration is defined by death due to a combination of factors including cognitive dysfunction and refusal of eating and drinking
